# Evaluation on the Seal Performance of SMP-Based Packers in Oil Wells

**DOI:** 10.3390/polym14040836

**Published:** 2022-02-21

**Authors:** Naihan Chen, Xuelin Dong, Yinji Ma

**Affiliations:** 1 Key Laboratory of Petroleum Engineering, China University of Petroleum, Beijing 102249, China; naihan_chen@163.com; 2 AML, Department of Engineering Mechanics, Tsinghua University, Beijing 100084, China; mayinji@tsinghua.edu.cn

**Keywords:** shape memory polymers, packers, sealability, contact stress, shear stress

## Abstract

Packers based on shape memory polymers (SMPs) are an emerging technology that have the advantages of compact structure, easy manufacture, and adaptability to complex wells. This paper proposes a finite element model to simulate the setting process and mechanical response of an SMP packer. The investigated material is an epoxy-based thermal responsive SMP, whose relaxation modulus and thermal expansion coefficient were measured at different temperatures. Based on the experimental data, the model describes the viscoelastic behavior of the SMP using the generalized Maxwell model. The results show that the SMP packer could provide sufficient contact stress under downhole conditions, even after the stress was relaxed. A further parametric study revealed that the most significant factor in sealing effects is the wellbore pressure, followed by the interference between the packer and the annular, the seal length, the pre-compression, and the setting temperature. High downhole pressures require more significant contact stress and increase the risk of slip between the packer and casing wall by promoting shear stress. Increasing the seal length and interference enhances the contact stress and mitigates the shear stress to improve the seal performance. Pre-compression and setting temperatures are minor factors that have little influence on sealability.

## 1. Introduction

Shape memory polymers (SMPs) are a class of intelligent materials that store a temporary shape and recover their original form when exposed to external stimuli, such as temperature, magnetism, radiation, and light [1,2,3,4]. Compared to shape memory alloys, SMPs have the advantages of low density, excellent mechanical properties, and easy manufacturing [5]. Hence, they have been applied to various fields, including aerospace structures [6], biomedical apparatus [7], and 3D or 4D printing [8], etc.

**Figure 1:**
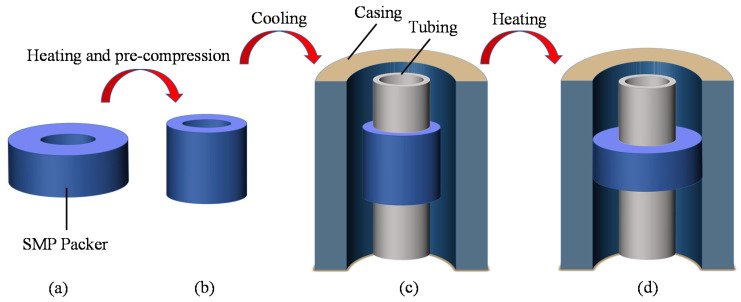
Setting process of a shape memory polymer packer: (**a**) the original shape of the shape memory polymer (SMP) packer, (**b**) after pre-compression, (**c**) run in the wellbore, (**d**) recovery to seal the annular by re-heating.

SMPs also have a variety of applications in petroleum engineering. Thermally actuated SMPs are the most commonly used materials, for example, intelligent plugging drilling fluids [9], self-expanding cement [10,11], intelligent sand control technology [12,13], etc. Field tests and laboratory studies have demonstrated that technologies involving SMPs have significantly improved oil recovery rates [14]. Recently, a packer made of thermo-responsive shape memory polymers with a smaller size has been proposed as a potential seal component [15,16]. Packers are an important downhole tool for sealing the annulus and isolating the oil and gas layers to implement production operations [17]. However, conventional mechanical packers have poor reliability, large size, difficult downhole access in complex wells, and high construction risks [18,19]. In contrast, packers made of thermally actuated shape memory polymers have a compact design, easy processing, and flexible adaptability [20]. Figure 1 depicts the working principle of the SMP packer. The packer cartridge in the initial shape is heated above its transition temperature (*T*_g_) and compressed or stretched to reduce its thickness. This programmed shape is fixed after cooling well below *T*_g_. The SMP packer then runs in the well with the mandril. Finally, it is activated by a heater to expand and seal the wellbore at the specified position.

The seal performance after setting is directly related to the effects of fracturing or other production operations [21]. A series of studies was conducted on conventional mechanical packers [22,23,24,25]. The majority of studies focused on the mechanical properties of the sealing element, that is, the elastomer [26,27,28]. The primary properties are elastic modulus, tensile strength, elongation at break, stress relaxation, etc. [29,30,31]. Because it is costly to conduct field tests to evaluate the performance of packers, many researchers have investigated their sealability based on finite element analysis (FEA) using experimentally obtained material parameters [32]. They chose the contact stress at the sealing interface as a critical indicator of the seal quality. Parametric studies on the seal length, packer thickness, interference or compression ratio, temperature, and wellbore pressure revealed the influence of these structural or downhole parameters on the contact stress. The results varied depending on the sealing element used. For instance, a conventional rubber packer induces uniformly distributed contact stress at the interface. The contact stress reaches a peak at the center of the seal length for a swellable elastomer packer [33]. Theoretical studies have demonstrated that the seal thickness has little effect on the contact stress for longer packers [34]. However, an FEA simulation indicated that the radial seal thickness plays a significant role in sealing [35]. Therefore, it is necessary to investigate the structural performance when a novel sealing element is applied.

SMP packers work utterly differently from conventional packers (Figure 1). It is imperative to investigate the mechanical behavior of an SMP packer for engineering applications. However, research on this area is still in its infancy. Baker Hughes [15,36] filed several patents of SMP packers with different structures, but their sealability was not evaluated publicly. Tong et al. developed a new enhanced thermo-actuated SMP composite and established a constitutive model to describe its mechanical behavior [20]. They carried out a numerical simulation and an experimental study on the packer element, which indicated that the contact stress provided by shape recovery would meet the requirements of the oil industry. Although not as a packer, SMP has been applied in fields for sand control in offshore wells. Fuxa et al. reported that this innovative approach could integrate wellbore isolation and selective production without increasing pumping complexity [37]. Despite the above work, further studies on SMP packers are needed to enhance their sealability.

This study proposed a finite element model to evaluate the seal performance of an SMP packer under subsurface conditions. The viscoelastic properties and thermal expansion coefficient of an epoxy-based thermal-responsive SMP were experimentally determined. Based on the time–temperature superposition principle, we constructed the master curve of the relaxation modulus according to the Williams–Landel–Ferry (WLF) equation [38]. This work utilizes the generalized Maxwell model and Prony series to characterize the viscoelastic behavior and shape recovery of the SMP packer. The proposed model successfully simulates the setting process of an SMP packer, upon which the effects of primary parameters on the contact and shear stresses at the sealing interface are investigated, including the packer length, interference, pre-compression, setting temperature, and wellbore pressure.

## 2. Experimental Characterization of the Polymer

**Figure 2:**
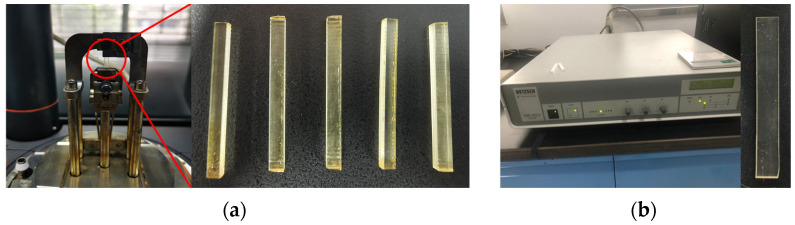
Specimens and test machine for the stress relaxation experiments and thermo-mechanical analysis: (**a**) relaxation test; (**b**) thermo-mechanical analysis.

In this work, we measured the relaxation modulus and thermal expansion coefficient of an epoxy-based thermally responsive SMP at different temperatures. The test selected a polymer with a glass transition temperature (*T*_g_) of 80 °C as reported by the manufacturer, accommodating the downhole condition. Figure 2 shows the samples that the shorter one (11.85 mm × 2.83 mm × 3.01 mm) is cut for stress relaxation test and the longer one (49.80 mm × 7.28 mm × 2.86 mm) for thermo-mechanical analysis.

**Figure 3:**
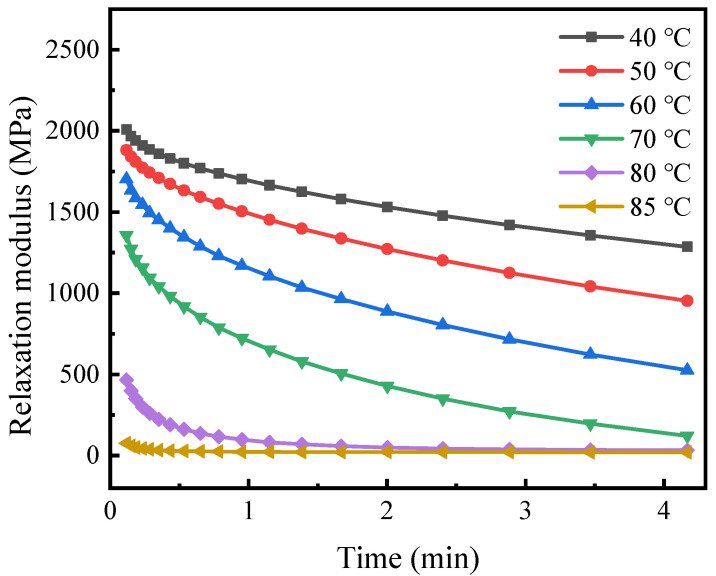
Relaxation modulus versus time under varying isothermal conditions.

It has been reported that the shape recovery effect of SMPs can be simulated by its relaxation modulus [39]. Thus, the current work measured the properties of the polymer using the instrument DMA Q800. The test temperature ranged from 40 °C to 80 °C in steps of 10 °C. A temperature of 85 °C was added to characterize the mechanical behavior above *T*_g_. The chamber first heated the specimen to the pre-set temperature and held it for 10 min. Then, the machine stretched the sample 0.11 mm, applying a nominal strain of approximately 0.1% for 257 s. Owing to the viscoelastic behavior, the tensile stress in the polymer decreased, and the corresponding modulus was recorded during the test. Figure 3 illustrates the relaxation moduli obtained at elevated temperatures. These curves show that the modulus drops considerably above *T*_g_.

**Figure 4:**
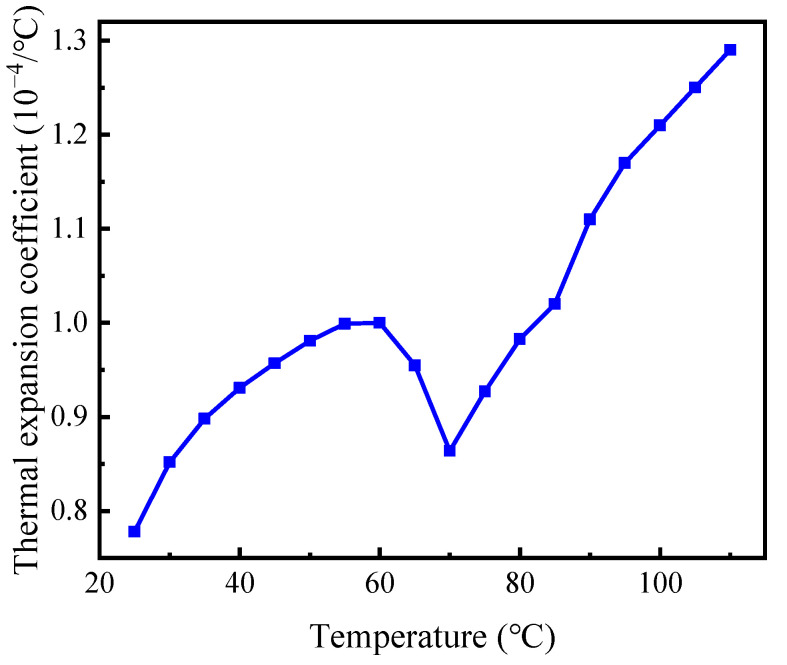
The thermal expansion coefficient at different temperatures.

The thermal expansion coefficient has a significant effect on the sealing performance of the SMP packer. We carried out a thermo-mechanical analysis of the polymer with a DIL-402EP thermal expansion instrument. A small tension (0.05 N) was applied to the specimen at 20 °C to keep it straight. The machine then elevated the temperature to 110 °C in steps of 5 °C. Monitoring the variation in length with temperature provided the thermal expansion coefficient, as shown in Figure 4. The non-monotonic curve indicates that the thermal expansion coefficient increases with the temperature first, drops deeply at 70 °C, and rises again. This unusual phenomenon may be explained by the “free volume theory” [40], and similar experimental observation is reported in relevant studies [41]. It is worth noting that a high temperature (>*T*_g_) induces significant thermal expansion. Because it often encounters high temperatures in the subsurface, it is necessary to consider thermal deformation to design an SMP packer.

## 3. Viscoelastic Parameters for SMP

Generally, the mechanical response of materials or structures is governed by the stress equilibrium equation (neglecting the body force) [42]:

(1) $$\frac{\partial \sigma_{ij}}{\partial x_{j}}=0,$$

where *σ_ij_* represents the stress components with *i*, *j* ∈ {1, 2, 3}, and *x_j_* is the coordinate. For linear, isotropic, and elastic materials, the stress is related to the strain with constitutive equations (incorporating the thermal strain):

(2) $$\sigma_{ij}=\frac{E}{1+\nu }\varepsilon_{ij}+\frac{E\nu }{\left(1+\nu )(1-2\nu \right)}\varepsilon_{kk}\delta_{ij}-\frac{E}{1-2\nu }\alpha \Delta T\delta_{ij},$$

where *ε_ij_* denotes the strain components, *ε_kk_* = *ε*_11_ + *ε*_22_ + *ε*_33_ is the volumetric strain, *δ_ij_* is the Kronecker delta, *E* and *ν* are Young’s modulus and Poisson’s ratio, respectively, *α* is the material’s thermal expansion coefficient, and Δ*T* represents the temperature difference between the current state and the initial state. Equation (2) indicates that the elastic modulus and thermal expansion coefficient are crucial to characterize the deformation and stress of materials. Instead of the simple linear form of Equation (2), the constitutive equation for viscoelastic materials is a hereditary integral [43]:

(3) $$\sigma =\int_{0^{-}}^{t}E(t-\tau )\frac{d\varepsilon (\tau )}{d\tau }d\tau ,$$

where *t* represents time and *E* is the tensile relaxation modulus.

**Figure 5:**
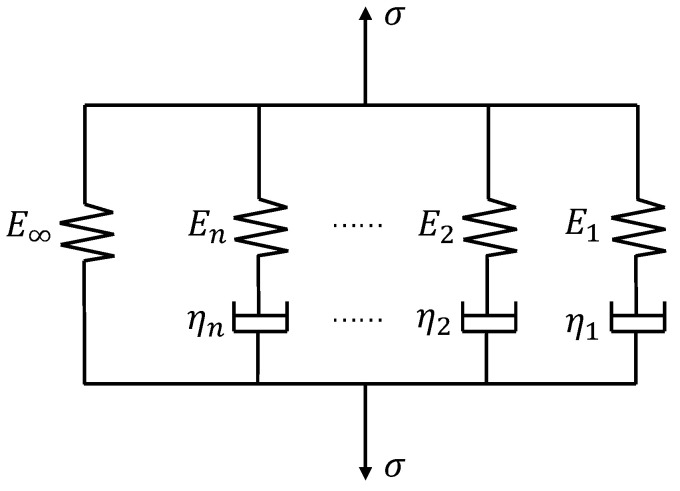
A schematic representation of the generalize Maxwell model.

Section 2 has obtained the relaxation modulus and thermal expansion coefficient of the SMP at different temperatures. To predict the thermo-mechanical behavior of shape memory polymers, we used the generalized Maxwell model to describe the viscoelastic response [44]. This model contains multiple parallel spring-dashpot elements, as shown in Figure 5, and can characterize the stress relaxation of polymers over a wide range of time and temperature. A Prony series can express the effective modulus of the generalized Maxwell model as follows [39]:

(4) $$E(t)=E_{\infty }+\sum_{i=1}^{N}E_{i}e^{-t/\tau_{i}},$$

where *E*_∞_ represents the equilibrium modulus of the material at which it has fully relaxed, *E_i_* and *τ_i_* are the elastic modulus and relaxation time of the *i*th element, respectively, *τ_i_* = *η_i_*/*E_i_* with *η_i_* denoting the viscosity of the *i*th dashpot, and *N* is the element number.

Equation (4) defines an *E*–*t* curve, which is referred to as the master curve. Usually, the master curve extends over a wide time range in a few decades. From an experimental viewpoint, it is practical to measure a set of *E*–*t* segments at different constant temperatures and then shift these segments along the logarithmic time scale to form a master curve at a reference temperature. This procedure is based on the time–temperature superposition principle, according to which the relaxation modulus at a particular temperature and time is related to that under other conditions as [45]:

(5) $$E(t,T_{\mathrm{ref}})=E(a_{T}t,T),$$

where *T*_ref_ is the reference temperature, *T* represents an arbitrary temperature, and *a_T_* is the time–temperature superposition shift factor. Equation (5) makes it possible to predict the viscoelastic behavior of polymers at different temperatures from the master curve. Naturally, *a_T_* depends on the temperature and is given by the WLF equation [45]:

(6) $$\log_{10}(a_{T})=-\frac{C_{1}(T-T_{\mathrm{ref}})}{C_{2}+T-T_{\mathrm{ref}}},$$

where *C*_1_ and *C*_2_ are material constants that can be extracted from the stress–relaxation experiment.

**Figure 6:**
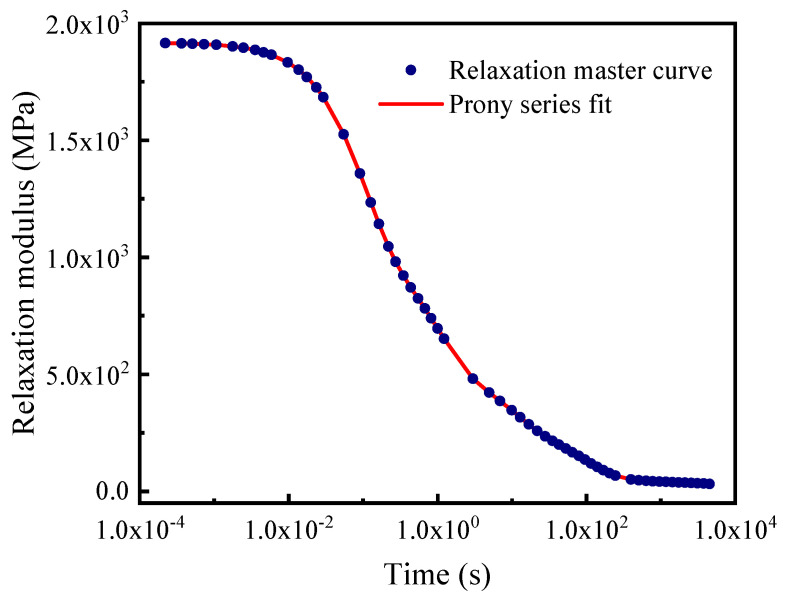
The relaxation master curve at the reference temperature.

The above segment shifting is subjective in that a set of experimental data may not always yield the same master curve. A mathematical algorithm from Gergesova et al. is adopted to construct the master curve, eliminating the manual shifting procedure [46]. It proposed an overlapping window to determine the datum point between two adjacent segments. For the current polymer used, the fitting parameters were *C*_1_ = 7.38 and *C*_2_ = 100.24 °C. The obtained master curve at *T*_g_ is shown in Figure 6, from which the parameters of the Prony series in Equation (4) are extracted, as shown in Table 1.

## 4. Simulation Modeling for the Setting Process

Setting an SMP packer contains some primary steps as follows:

1. Increasing the temperature above *T*_g_ significantly reduced the modulus of the SMP packer. Then, a radial compressive load is applied to the packer to extend its axial length and reduce its outer diameter. The compressed packer clings firmly to the mandril, making it easier to run in the wellbore.
2. Keep the compressive load and decrease the temperature well below *T*_g_.
3. Slowly release the load, and the SMP packer store the compressed shape.
4. The packer was lowered into the well. When it arrives at the setting position, it is heated again above *T*_g_ using a downhole heater. Finally, the packer gradually returns to its original shape. Then, it expands to contact the inner wall of the casing or the formation, which produces contact stress to achieve sealing.

The simulation is supposed to mimic the setting process as described above.

### 4.1. Material Parameters in FEA

This work implements a simulation using the commercial finite element analysis software ABAQUS. It defines the viscoelastic material with the Prony series in a slightly different form [47]:

(7) $$g_{R}(t)=1-\sum_{i=1}^{N}g_{{}_{i}}\left(1-e^{-t/\tau_{{}_{i}}}\right),$$

where *g_R_* is the ratio of the shear relaxation modulus to the instantaneous modulus, *g_i_* is the Prony coefficient, and *τ_i_* is the relaxation time. This study assumes that the SMP is incompressible, which makes it easy to transform *E_i_* to *g_i_* from Equation (4) as:

(8) $$g_{i}=\frac{E_{{}_{i}}}{E_{{}_{0}}},$$

where *E*_0_ is the instantaneous tensile relaxation modulus, which can be obtained from Equation (4):

(9) $$E_{0}=E(0)=E_{\infty }+\sum_{i=1}^{N}E_{{}_{i}}$$

### 4.2. The Geometry of the Model

The simulation assumes that the packer would seal an annular space between the tubing and casing. We selected a typical outer diameter of 73.00 mm for the tubing and an inner diameter of 124.26 mm for the casing. Accordingly, the inner diameter of the SMP packer was set equal to the outer diameter of the tubing. To fulfill the sealing, the original thickness of the packer should be thicker than the annular width. The difference between the outer diameter of the packer and the inner diameter of the casing determines the magnitude of the interference, which is critical to seal performance. This model first sets the packer’s outer diameter as 130 mm and then changes it to obtain different interferences.

**Figure 7:**
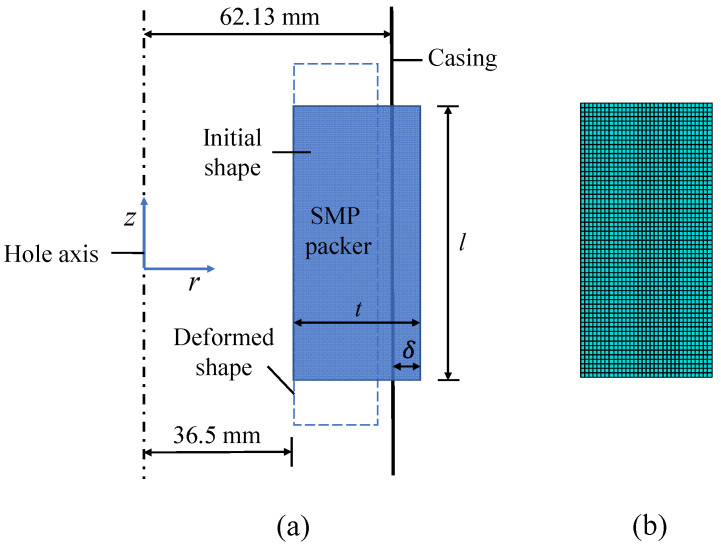
A schematic of the model and meshing: (**a**) the model components, (**b**) the mesh.

Figure 7a shows that the packer’s geometry and the load and boundary conditions are axisymmetric. Then, this work constructed a two-dimensional plane, axisymmetrical model. The casing is deemed a rigid body. The tubing is replaced by varying the boundary conditions on the left side of the packer during each step of setting, simplifying the simulation. The model meshed the packer with 2013 solid 4-node quadrilateral brick elements (CAX4R), as shown in Figure 7b.

### 4.3. Simulation Steps and Boundary Conditions

Each step of the setting process corresponds to the specific temperature and boundary conditions. Considering the stress relaxation effect, the current model contains seven steps, including one initial step for the original shape of the packer, five viscoelastic steps for the packer setting, and one general static step for applying wellbore pressure. The detailed settings are as follows:

1. The initial step defines the temperature field as 120 °C (>*T*_g_) and constrains the radial displacement on the left side of the packer.
2. The first viscoelastic step applies a radial displacement (−10 mm in the radial direction) on the right side to compress the packer under the constant temperature defined in the initial step.
3. In the second viscoelastic step, the field temperature decreased to 50 °C with compression.
4. During the third viscoelastic step, the model keeps the low temperature unchanged and releases the displacement load. Meanwhile, the rigid casing moves radially to form an annular gap between the tubing and casing.
5. The fourth viscoelastic step increases the temperature again above *T*_g_ to activate shape recovery.
6. Considering the stress relaxation of polymers, the fifth step holds the temperature for a while until the contact stress between the packer and the casing reaches a steady value.
7. Finally, the extra general step applies pressure (20 MPa) to the top edge of the packer. Under subsurface conditions, the packer must withstand the pressure difference between the annular and pore fluids. In an extreme case, only one end of the packer is subjected to wellbore pressure.

**Figure 8:**
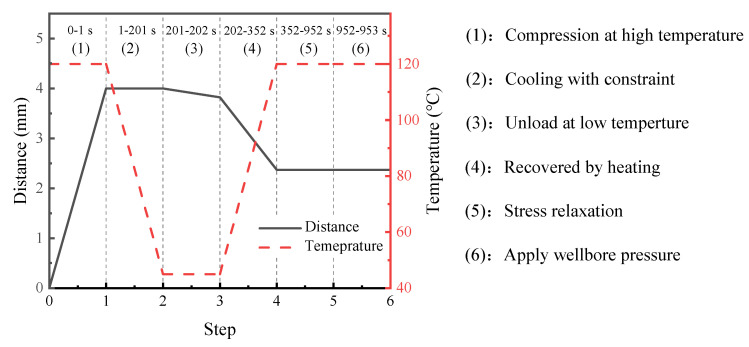
Displacement boundary at the right side of the packer and temperature field of the model in each analysis step.

Figure 8 illustrates the displacement boundary on the right side of the packer and the field temperature of the model at each step. The first and third viscoelastic steps and the final general static step were set to 1 s because of the constant temperature at these steps to save computation time. The second and fourth viscoelastic steps corresponded to the cooling and heating processes, and lasted for 100 s and 200 s, respectively. In addition, the fifth viscoelastic step has a computation time of 600 s to ensure that the stress is fully relaxed. It should be noted that during the third analysis step, the radial restraint on the right side of the packer was released. The displacement data in and after this step in Figure 8 are based on the calculation results. Apart from the axisymmetric condition for the simulation, the current model contains other assumptions, including the linear viscoelastic response of the polymer, the incompressibility, and the Coulomb friction between the polymer and the casing with a friction coefficient of 0.2.

## 5. Results and Discussions

During the setting process, the shape memory polymer packer expands to contact the casing wall or the borehole activated by heating, creating contact stress as a barrier to the wellbore. The magnitude and distribution of the contact stress are crucial to the sealing capacity of the packer. Intuitively, greater and more uniformly distributed contact stresses lead to a better seal quality. The wellbore pressure pushes the packer to move axially, inducing shear stresses at the sealing interface. Excessive shear stress makes the packer prone to slip along the interface. Therefore, the contact stress characterizes the sealability, while the shear stress identifies the risk of slip failure, which are two essential indicators for evaluating the seal performance of packers.

### 5.1. Base Case Simulation for the Seal Performance

**Figure 9:**
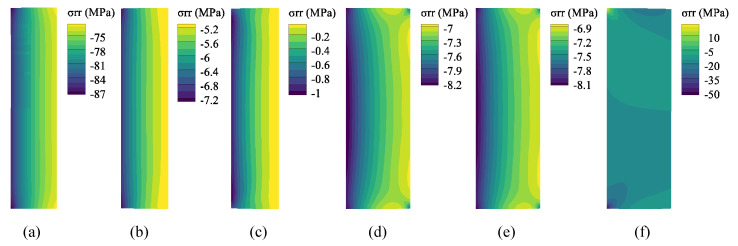
Radial stress of the SMP packer for each step: (**a**) Step 1, (**b**) Step 2, (**c**) Step 3, (**d**) Step 4, (**e**) Step 5, (**f**) Step 6.

Figure 9 shows the radial stress of the SMP packer obtained in the analysis steps. In Step 1, after the packer is compressed from its original shape at a high temperature above *T*_g_, the radial stress increases and reaches a maximum of 87 MPa. This stress component gradually decreases during cooling and unloading (Steps 2 and 3), although the material modulus increased as the temperature decreased. In the heating stage (Step 4), the SMP packer expands to contact the casing wall owing to shape recovery and thermal expansion. Then, the contact stress is generated at the interface between the packer and casing (the right side of the packer) with high values at both ends and relatively low but uniform values in the middle. The magnitude of the contact stress is in the range of 6.9~8.5 MPa, similar to Tong et al., which validates the numerical simulation [20]. As expected, the contact stress decreased owing to the stress relaxation in Step 5. Finally, the packer expands laterally under a wellbore pressure of 20 MPa at the top, increasing the contact stress to approximately 15 MPa.

**Figure 10:**
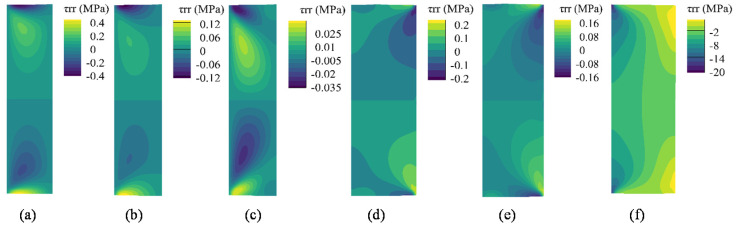
Shear stress of the SMP packer for each step: (**a**) Step 1, (**b**) Step 2, (**c**) Step 3, (**d**) Step 4, (**e**) Step 5, (**f**) Step 6.

As the packer undergoes mainly radial deformation during the setting process, there is little shear stress at the sealing interface in Steps 1–5, as shown in Figure 10. After the wellbore pressure was applied, a remarkable amount of shear stress (approximately −2 MPa) was generated at the interface. Most sealing interfaces have negative shear stress. The current coordinate system (see Figure 7) defines the downward (upward) shear stress with negative (positive) values. This distribution implies that the middle part of the SMP packer is more likely to slip.

### 5.2. Effects of Seal Length, Interference, Pre-Compression, Temperature, and Wellbore Pressure

Various parameters affect the seal performance of the SMP packer, including the seal length (*l*), the interference (*δ*), the pre-compression (*c*), the setting temperature (*T*_s_), and the wellbore pressure (*p*_w_). The base case simulation shows that the contact stress occurs only in Steps 4–6. Hence, we repeated the model simulation with the above parameters to obtain the contact and shear stresses at the packer–casing interface in these steps. The simulation changes one parameter and fixes the others in one calculation.

**Figure 11:**
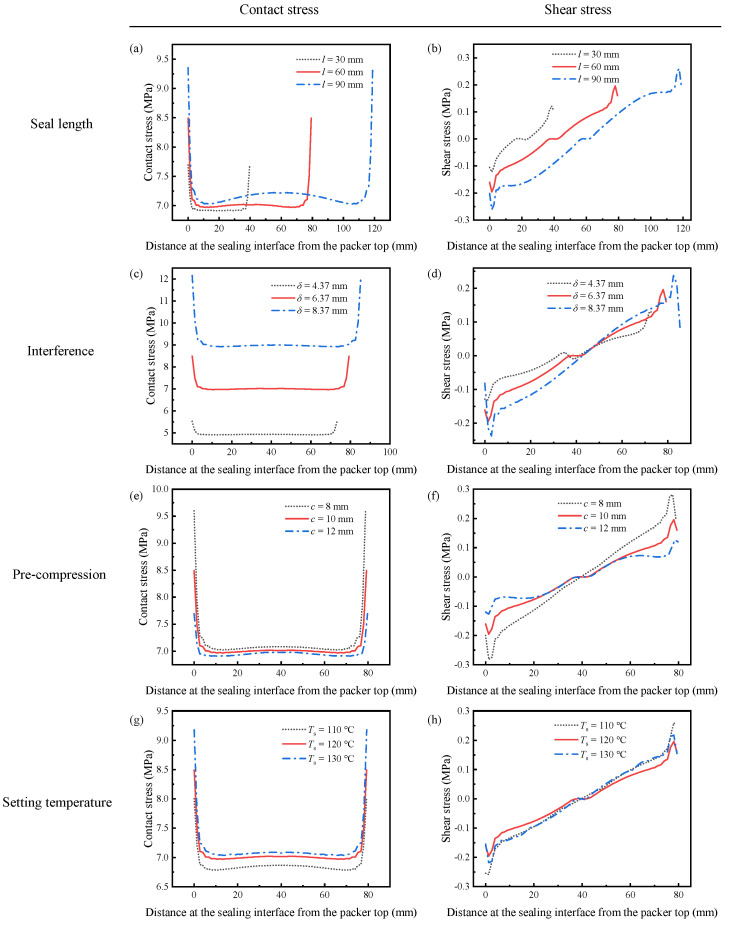
Distribution of the contact and shear stress after shape recovery under various sealing conditions: (**a**) contact stress with different seal lengths, (**b**) shear stress with different seal lengths, (**c**) contact stress with different interferences, (**d**) shear stress with different interferences, (**e**) contact stress for different pre-compression, (**f**) shear stress for different pre-compression, (**g**) contact stress at different setting temperatures, (**h**) shear stress at different setting temperatures.

**Figure 12:**
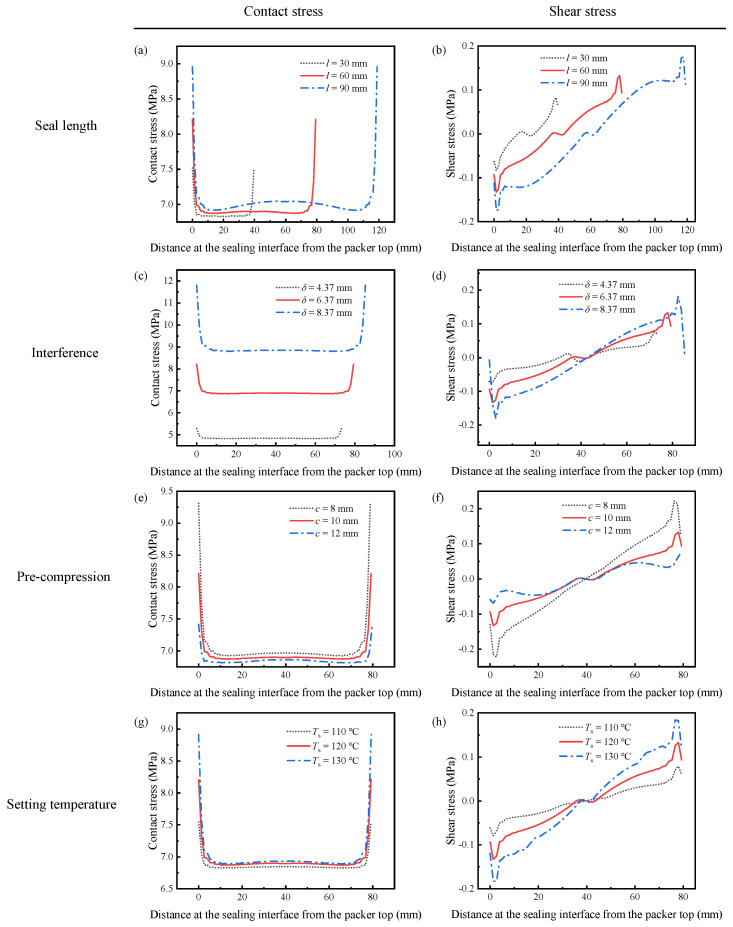
The contact and shear stress after relaxation under different sealing parameters: (a) contact stress with different seal lengths, (**b**) shear stress with different seal lengths, (**c**) contact stress with different interferences, (**d**) shear stress with different interferences, (**e**) contact stress for different pre-compression, (**f**) shear stress for different pre-compression, (**g**) contact stress at different setting temperatures, (**h**) shear stress at different setting temperatures.

**Figure 13:**
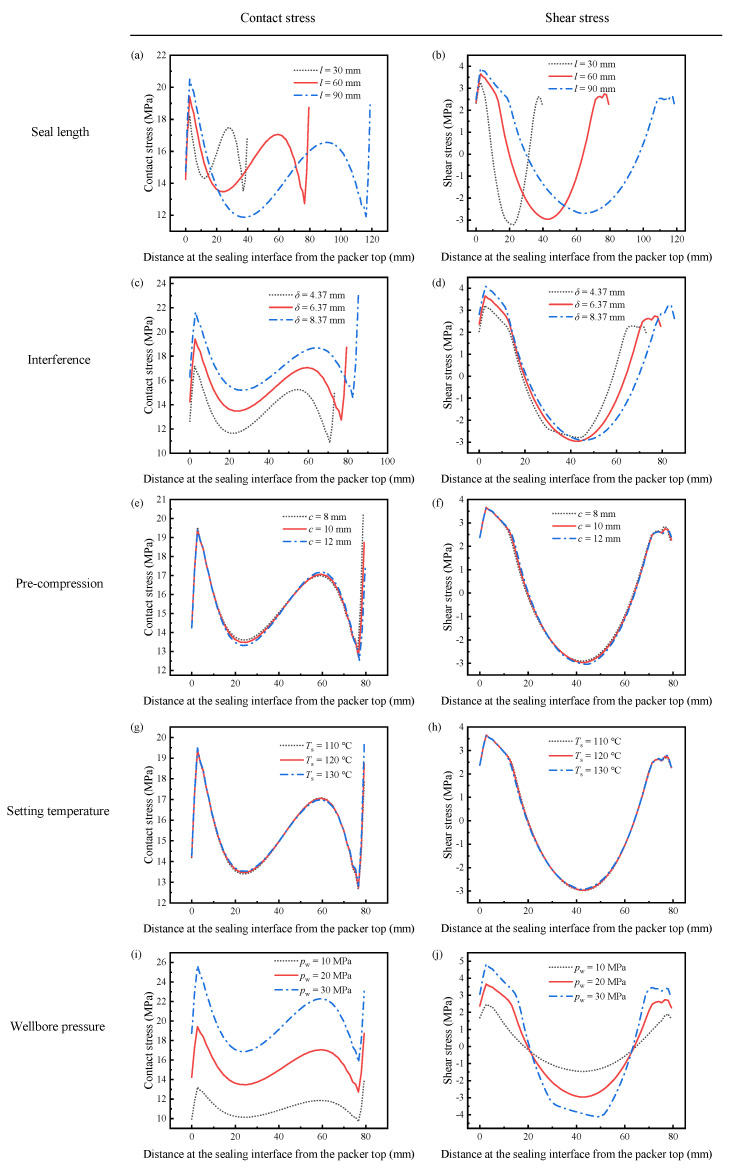
The contact and shear stress when the wellbore pressure is applied to the packer: (**a**) contact stress with different seal lengths, (**b**) shear stress with different seal lengths, (**c**) contact stress with different interferences, (**d**) shear stress with different interferences, (**e**) contact stress for different pre-compression, (**f**) shear stress for different pre-compression, (**g**) contact stress at different setting temperatures, (**h**) shear stress at different setting temperatures, (**i**) contact stress under various wellbore pressure, (**j**) shear stress under various wellbore pressure.

Figure 11a, Figure 12a and Figure 13a reveal the influence of the seal length on the seal performance after shape recovery, stress relaxation, and the application of the wellbore pressure, respectively. Figure 11a and Figure 12a indicate that the contact stress decreases after the polymer completely relaxes. In addition, a longer seal length generates more significant contact stress with a more non-uniform distribution in the middle of the packer. As the packer becomes longer, it accumulates more shrinkage in the axial direction during shape recovery, which makes its middle part more compressive. However, the longer packer exhibited lower contact stress when the wellbore pressure was applied to the rubber top (Figure 13a). Owing to the wellbore pressure, the lateral expansion is minor for a longer packer, which decreases the contact stress.

Figure 11b and Figure 12b show the shear stress distribution of different seal lengths in Steps 4 and 5, respectively. The packer produces very little shear stress during the setting because most of the deformation is radial. Longer packers induced greater shear stress. As shown in Figure 13b, the shear stress increases significantly when the packer is subjected to pressure. The increased shear stress fluctuated along the interface, with positive values at both ends and negative values in the middle. It is worth noting that increasing the seal length may mitigate shear stress in the middle part.

Compared to the working principle of conventional packers, SMP packers require their initial thickness to be larger than the annular space between the tubing and casing or between the casing and wellbore. This interference is supposed to generate sufficient contact stress to form a seal. Therefore, interference is a critical parameter for evaluating the seal performance. This study considers three different interferences as 4.37 mm, 6.37 mm, and 8.37 mm.

Figure 11c and Figure 12c show the contact stress with various interferences during setting and stress relaxation. The amount of interference had a significant influence on the contact stress. A 31.4% increase in *δ* (from 6.37 mm to 8.37 mm) enlarges the contact stress by 28.6% (7.0 MPa to 9.0 MPa). More significant interference compresses the packer more deeply. This high contact stress was maintained after the wellbore pressure was exerted on the top of the packer, as shown in Figure 13c. However, increasing *δ* requires thicker packers, which would cost more.

The shear stress is still insignificant when the packer recovers owing to shape memory effects, as depicted in Figure 11d and Figure 12d. In addition, more interference leads to greater shear stress. The plots in Figure 13d show that the shear stress at both ends of the packer increases with the interference under wellbore pressure. Specifically, the interference of 8.37 mm induces the highest shear stress of 4 MPa, while for *δ* = 4.37 mm, the maximum shear stress is 3 MPa. Most of the shear stresses at the interface under different interferences are close to each other. Increasing *δ* improves seal performance by providing high contact stress without aggravating slip failure.

As shown in Figure 1, the SMP packer should be precompressed before setting. Compressing the packer in the radial direction could reduce its radius and elongate its length, making it easier to run in the wellbore. Then, the pre-compression has a considerable influence on the recovery expansion of the shape-memory polymer, thus affecting the seal performance. The FEA model takes three different compression amounts of 8 mm, 10 mm, and 12 mm to study its effect on the setting. Figure 11e, Figure 12e and Figure 13e indicate that the contact stress under different pre-compressions during shape recovery, stress relaxation, and wellbore pressure are very close to each other. Notably, the three lines in Figure 13e nearly overlap. For example, the contact stresses in the middle of the packer obtained at the end of shape recovery (Figure 11e) with pre-compressions of 8 mm, 10 mm, and 12 mm are 7.08 MPa, 7.01 MPa, and 6.98 MPa, respectively. Because shape memory polymers can restore their initial shape, different pre-compressions would induce similar interferences between the packer and the annular. Figure 11e and Figure 12e show that a greater pre-compression would result in a smaller contact stress in Steps 4 and 5. This decrease may be attributed to the imperfect shape recovery that the polymer expands less when it is compressed more.

As shown in Figure 11f, Figure 12f and Figure 13f, the pre-compression had little impact on the shear stress. From Figure 11f and Figure 12f, the greater the pre-compression, the smaller the shear stress. It seems that pre-compression is a trivial factor in the seal performance of an SMP packer. However, when the pre-compression exceeds the yield strength of the polymer, the packer would lose its shape memory function and fail the setting process.

The setting temperature is also an essential parameter for the recovery efficiency of shape-memory polymers. The current simulation reveals that a higher setting temperature produces greater contact stress during shape recovery, as illustrated in Figure 11g. Specifically, when the setting temperature increased from 110 °C to 130 °C, the contact stress in the middle increased by 2.9%. This increase hinges on thermal expansion at higher setting temperatures. Unexpectedly, Figure 12g shows that stress relaxation would eliminate the difference in the contact stress between various setting temperatures, which leads to indistinguishable lines in Figure 13g.

Figure 11h and Figure 12h illustrate the shear stress distribution under different setting temperatures in Steps 4 and 5, respectively. Unlike the contact stress, the plots are similar during shape recovery (Figure 11h) but disperse after stress relaxation (Figure 12h). The packer generates a more significant shear stress at the interface under a higher setting temperature. When the packer is subjected to the wellbore pressure, the shear stress plots under different setting temperatures almost coincide (Figure 13g) as the contact stress does. This indicates that the setting temperature has an insignificant influence on seal performance.

The contact stress distributions under different wellbore pressures were similar, as shown in Figure 13i, and their values increased with the wellbore pressure. As *p*_w_ increased from 10 MPa to 30 MPa, the maximum contact stress increased from 14 MPa to 26 MPa. A tremendous axial pressure would cause a more lateral expansion in the packer, inducing more significant contact stress. Figure 13j has the same trend whereby the maximum shear stress in the middle of the packer rises from 1.46 MPa to 4.12 MPa when the wellbore pressure changes from 10 MPa to 30 MPa. The enhanced shear stress increases the likelihood of slip failure.

### 5.3. Sensitivity Analysis

**Figure 14:**
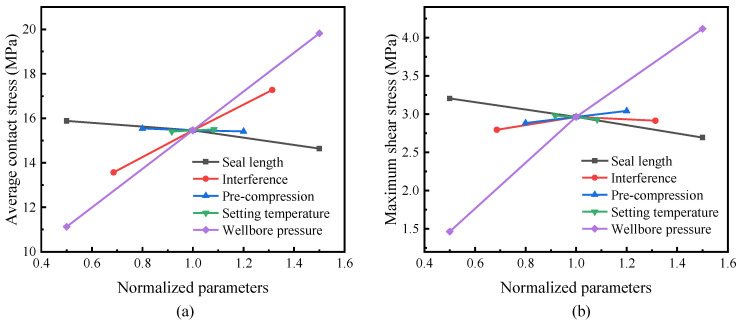
Sensitivity of the contact and shear stress to various parameters: (**a**) the average contact stress; (**b**) the shear stress.

This study normalizes the five parameters by the base case to reveal the contact and shear stress sensitivity to them, as listed in Table 2. For simplicity, the average contact stress over the interface is defined as:

(10) $${\bar{\sigma }}_{C}=\frac{1}{l}\int_{-l/2}^{l/2}\sigma_{C}dz,$$

where ${\bar{\sigma }}_{c}$ represents the average contact stress. Taking the normalized parameter as the abscissa, the average contact stress, and the maximum shear stress in the middle of the packer under the wellbore pressure as ordinates, we constructed a sensitivity diagram, as shown in Figure 14. The greater the absolute slope of the curve, the more sensitive the average contact stress and shear stress to the corresponding parameters. From Figure 14, the most influential contact and shear stress parameters are the wellbore pressure, followed by the interference and seal length. The curves for the pre-compression and setting temperatures are nearly horizontal, which means that they are insignificant.

A higher wellbore pressure usually corresponds to deep wells, which are dependent on subsurface conditions. In terms of design, it is practical to adjust the length and interference of the packer to adapt it to specific circumstances. Remarkably, a greater wellbore pressure requires a high contact stress to seal the annular, but it also causes significant shear stress at the interface. According to Figure 14, increasing the interference could provide substantial contact stress, while a longer packer would reduce the shear stress. Hence, it is advisable to apply a longer packer to compensate for the shear stress increase caused by a larger interference or a greater wellbore pressure.

## 6. Conclusions

In this study, a finite element model was proposed to investigate the mechanical behavior and critical parameters controlling the seal performance of a packer based on shape memory polymers under subsurface conditions. Combined with the viscoelastic characteristics obtained experimentally from stress relaxation tests and thermo-mechanical analysis, the model could mimic the shape recovery and mechanical response of the SMP packer during setting. Based on the current model, a parametric study was carried out to analyze the effects of pertinent parameters on the seal performance, including the seal length, interference, pre-compression, setting temperature, and wellbore pressure. The analysis adopted contact and shear stress as two critical indicators to represent the seal quality.

In a base case simulation, the model calculation indicates that the SMP packer can provide sufficient contact stress between the tubing and casing to seal the annular successfully. Even though the contact stress is reduced due to relaxation, its stable value satisfies the demand of the setting. In addition, there was little shear stress before the wellbore pressure was applied. When the packer is subjected to a pressure difference, a significant amount of shear stress may occur at the interface, threatening the sealing reliability.

A further parametric study showed that a longer packer induces a greater but more non-uniformly distributed contact stress. Longer packers also help prevent slip between the packer and casing by reducing the shear stress in the direction of the wellbore pressure. The interference is crucial to the contact stress, and more interference could lead to a significant increase in the contact stress. In contrast, the interference had little effect on the shear stress. Reducing the pre-compression or increasing the setting temperature could increase the contact stress to a small extent. They had nearly no influence on the shear stress. A high wellbore pressure increases the contact stress and causes it to fluctuate along the interface. It also induces a high shear stress that poses a slip tendency in the middle part of the packer.

Finally, the sensitivity analysis indicated that the most influential parameter for the contact and shear stress for an SMP packer is the wellbore pressure, followed by the interference, seal length, setting temperature, and pre-compression. Optimizing the interference and packer length is more practical for obtaining reliable contact stress and appropriate shear stress from a design perspective. More attention should be paid to the pre-compression and setting temperature during the shape-recovery process to guarantee successful sealing.

## Figures and Tables

**Table 1:** Prony parameters extracted for the SMP.

i	τ_i_ (s)	E_i_ (MPa)
1	0.01	490.60
2	0.39	769.50
3	3.27	313.27
4	26.67	323.98
5	247.24	2.18
6	6.65 × 10^5^	35.31

**Table 2:** Normalized value of each parameter.

Parameters	Parameter Value (Normalized Value)		
Seal length (mm)	30 (0.5)	60 (1.0)	90 (1.5)
Interference (mm)	4.37 (0.69)	6.37 (1.0)	8.37 (1.31)
Pre-compression (mm)	8 (0.8)	10 (1.0)	12 (1.2)
Setting temperature (°C)	110 (0.92)	120 (1.0)	130 (1.08)
Wellbore pressure (MPa)	10 (0.5)	20 (1.0)	30 (1.5)
